# 3,6-Dimethyl-*o*-phenyl­enedimethanol

**DOI:** 10.1107/S1600536809053513

**Published:** 2009-12-19

**Authors:** Humaira Yasmeen Gondal, Muhammad Ali, Alain Krief, Muhammad Zia-ur-Rehman

**Affiliations:** a Department of Chemistry, University of Sargodha, Sargodha, Pakistan; b Laboratoire de Chimie Organique de Synthese, Facultés Universitaires Notre-Dame de la Paix, Rue de Bruxelles 61, B-5000 Namur, Belgium; c Applied Chemistry Research Centre, PCSIR Laboratories Complex, Ferozpure Road, Lahore 54600, Pakistan

## Abstract

The title compound, C_10_H_14_O_2_, synthesized by reduction of 4,7-dimethyl-2-benzofuran-1,3-dione, crystallizes with two independant mol­ecules in the asymmetric unit, both showing an intra­molecular O—H⋯O hydrogen bond. The crystal packing is stabilized by O—H⋯O hydrogen bonds.

## Related literature

For the influence of chelation to (semi-)metals on the geometry of bifunctional alcohols, see: Klüfers & Vogler (2007 ▶). For a related compound, see: Betz *et al.* (2009 ▶).
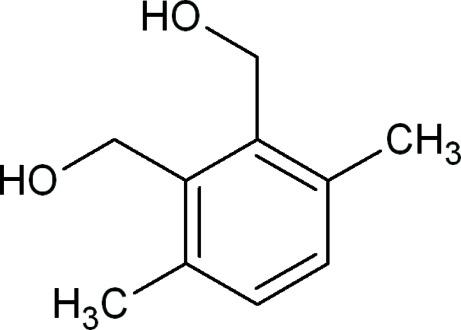


## Experimental

### 

#### Crystal data

C_10_H_14_O_2_
*M* *_r_* = 166.21
Monoclinic, 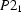

*a* = 9.5821 (7) Å
*b* = 8.7184 (7) Å
*c* = 11.7522 (9) Å
β = 107.810 (4)°
*V* = 934.73 (12) Å^3^
*Z* = 4
Mo *K*α radiation
μ = 0.08 mm^−1^
*T* = 296 K
0.33 × 0.10 × 0.07 mm

#### Data collection

Bruker APEXII CCD area-detector diffractometer
Absorption correction: multi-scan (*SADABS*; Sheldrick, 1997 ▶) *T*_min_ = 0.974, *T*_max_ = 0.994
10452 measured reflections
2468 independent reflections
1296 reflections with *I* > 2σ(*I*)
*R*_int_ = 0.041

#### Refinement

*R*[*F* ^2^ > 2σ(*F* ^2^)] = 0.047
*wR*(*F* ^2^) = 0.121
*S* = 0.99
2468 reflections
225 parameters
1 restraint
H-atom parameters constrained
Δρ_max_ = 0.11 e Å^−3^
Δρ_min_ = −0.12 e Å^−3^

### 

Data collection: *APEX2* (Bruker, 2007 ▶); cell refinement: *SAINT* (Bruker, 2007 ▶); data reduction: *SAINT*; program(s) used to solve structure: *SHELXS97* (Sheldrick, 2008 ▶); program(s) used to refine structure: *SHELXL97* (Sheldrick, 2008 ▶); molecular graphics: *ORTEP-3* (Farrugia, 1999 ▶) and *Mercury* (Macrae *et al.*, 2006 ▶); software used to prepare material for publication: *SHELXL97* and *PLATON* (Spek, 2009 ▶).

## Supplementary Material

Crystal structure: contains datablocks I, global. DOI: 10.1107/S1600536809053513/bt5136sup1.cif

Structure factors: contains datablocks I. DOI: 10.1107/S1600536809053513/bt5136Isup2.hkl

Additional supplementary materials:  crystallographic information; 3D view; checkCIF report

## Figures and Tables

**Table 1:** Hydrogen-bond geometry (Å, °)

*D*—H⋯*A*	*D*—H	H⋯*A*	*D*⋯*A*	*D*—H⋯*A*
O1—H1⋯O2^i^	0.82	1.89	2.706 (3)	174
O2—H2⋯O1	0.82	1.97	2.713 (4)	151
O3—H3⋯O4^ii^	0.82	1.90	2.709 (4)	167
O4—H4⋯O3	0.82	1.98	2.717 (4)	150

Symmetry codes: (i) 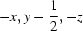; (ii) 
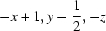
.

**Fig. 1.**
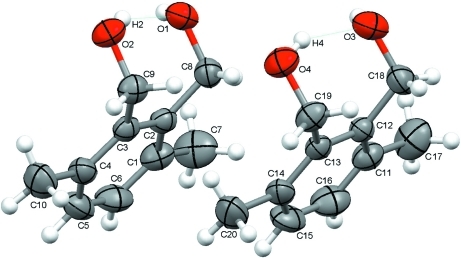
The molecular structure of (I), with displacement ellipsoids at the 50% probability level.

**Fig. 2.**
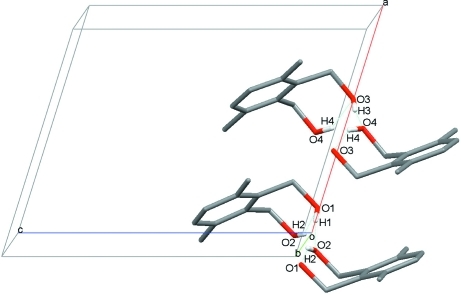
Perspective view of the three-dimensional crystal packing showing hydrogen-bonded interactions (dashed lines). H atoms not involved in hydrogen bonding have been omitted for clarity.

## supplementary crystallographic information

### Comment

In continuation of our work regarding the synthesis of various hetrocycles, structure of (3,6-dimethylbenzene-1,2-diyl) dimethanol   has been determined. Such diols may act as good ligands for the chelation of (semi-)metals (Klüfers & Vogler, 2007). Bond lengths and bond angles of the title molecule (Scheme 1; Fig. 1) are almost similar to those in the related molecules (Betz *et al.*, 2009).

Methyl groups at C7 & C10 are displaced by 0.71 (2)° and 0.85 (1)°, respectively, from the plane of the aromatic ring. Two independant asymmetric molecules exist in a unit cell and the benzylic oxygen of each molecule is involved in an intramolecular O—H···O hydrogen bond, forming a seven-membered hydrogen-bonded ring. Each molecule is centrosymmetrically linked to their adjacent ones through O—H···O hydrogen bonds   (Table 1; Fig. 2).

### Experimental

A mixture of 4,7-dimethyl-2-benzofuran-1,3-dione (0.176 g; 1.0 mmole), lithium aluminium hydride (0.042; 1.1 mmole), diethyl ether (20 ml) and tetrahydrofuran (20 ml) was refluxed for 15 h. Reaction mixture was than cooled and quenched with ice cooled water followed by the addition of aqueous sodium hydroxide (15%) to make the contents alkaline. Resulting solid was filtered off and washed with ether. Filtrate was concentrated to obtain (3,6-dimethylbenzene-1,2-diyl) dimethanol followed by its purification on silica gel column eluted by 60% diethyl ether in pentane.

### Refinement

All H atoms were identified in a difference map and then were treated as riding (O—H = 0.82, C—H = 0.93 or 0.97 Å), with *U*_iso_(H) = 1.2*U*_eq_(C,O). In the absence of significant anomalous dispersion effects, Friedel pairs were merged.

### Crystal data

**Table:**

C_10_H_14_O_2_	*F*(000) = 360
*M**_r_* = 166.21	*D*_x_ = 1.181 Mg m^−^^3^
Monoclinic, *P*2_1_	Mo *K*α radiation, λ = 0.71073 Å
Hall symbol: P 2yb	Cell parameters from 1602 reflections
*a* = 9.5821 (7) Å	θ = 3.0–19.6°
*b* = 8.7184 (7) Å	µ = 0.08 mm^−^^1^
*c* = 11.7522 (9) Å	*T* = 296 K
β = 107.810 (4)°	Needle, colorless
*V* = 934.73 (12) Å^3^	0.33 × 0.10 × 0.07 mm
*Z* = 4	

### Data collection

**Table:**

Bruker APEXII CCD area-detector diffractometer	2468 independent reflections
Radiation source: fine-focus sealed tube	1296 reflections with *I* > 2σ(*I*)
graphite	*R*_int_ = 0.041
φ and ω scans	θ_max_ = 28.5°, θ_min_ = 2.2°
Absorption correction: multi-scan (*SADABS*; Sheldrick, 1997)	*h* = −7→12
*T*_min_ = 0.974, *T*_max_ = 0.994	*k* = −11→11
10452 measured reflections	*l* = −15→15

### Refinement

**Table:**

Refinement on *F*^2^	Primary atom site location: structure-invariant direct methods
Least-squares matrix: full	Secondary atom site location: difference Fourier map
*R*[*F*^2^ > 2σ(*F*^2^)] = 0.047	Hydrogen site location: inferred from neighbouring sites
*wR*(*F*^2^) = 0.121	H-atom parameters constrained
*S* = 0.99	*w* = 1/[σ^2^(*F*_o_^2^) + (0.0538*P*)^2^ + 0.0276*P*] where *P* = (*F*_o_^2^ + 2*F*_c_^2^)/3
2468 reflections	(Δ/σ)_max_ < 0.001
225 parameters	Δρ_max_ = 0.11 e Å^−^^3^
1 restraint	Δρ_min_ = −0.12 e Å^−^^3^

### Special details

**Table:**

Geometry. All s.u.'s (except the s.u. in the dihedral angle between two l.s. planes) are estimated using the full covariance matrix. The cell s.u.'s are taken into account individually in the estimation of s.u.'s in distances, angles and torsion angles; correlations between s.u.'s in cell parameters are only used when they are defined by crystal symmetry. An approximate (isotropic) treatment of cell s.u.'s is used for estimating s.u.'s involving l.s. planes.
Refinement. Refinement of *F*^2^ against ALL reflections. The weighted *R*-factor *wR* and goodness of fit *S* are based on *F*^2^, conventional *R*-factors *R* are based on *F*, with *F* set to zero for negative *F*^2^. The threshold expression of *F*^2^ > 2σ(*F*^2^) is used only for calculating *R*-factors(gt) *etc*. and is not relevant to the choice of reflections for refinement. *R*-factors based on *F*^2^ are statistically about twice as large as those based on *F*, and *R*- factors based on ALL data will be even larger.

### Fractional atomic coordinates and isotropic or equivalent isotropic displacement parameters (Å^2^)

**Table:**

	*x*	*y*	*z*	*U*_iso_*/*U*_eq_	
C1	0.1958 (4)	0.2892 (4)	0.2857 (3)	0.0643 (10)	
C2	0.2068 (3)	0.4103 (4)	0.2113 (3)	0.0511 (8)	
C3	0.1604 (3)	0.5579 (4)	0.2305 (2)	0.0492 (8)	
C4	0.1010 (3)	0.5855 (4)	0.3226 (3)	0.0587 (9)	
C5	0.0928 (4)	0.4638 (5)	0.3960 (3)	0.0776 (12)	
H5	0.0555	0.4804	0.4593	0.093*	
C6	0.1379 (4)	0.3208 (5)	0.3776 (3)	0.0747 (11)	
H6	0.1297	0.2418	0.4283	0.090*	
C7	0.2426 (5)	0.1270 (4)	0.2686 (4)	0.0982 (14)	
H7A	0.2343	0.0635	0.3329	0.147*	
H7B	0.3426	0.1276	0.2679	0.147*	
H7C	0.1808	0.0872	0.1940	0.147*	
C8	0.2641 (3)	0.3817 (4)	0.1068 (3)	0.0619 (9)	
H8A	0.3412	0.4550	0.1094	0.074*	
H8B	0.3063	0.2797	0.1137	0.074*	
C9	0.1713 (3)	0.6851 (4)	0.1473 (3)	0.0590 (8)	
H9A	0.1653	0.7829	0.1849	0.071*	
H9B	0.2660	0.6798	0.1336	0.071*	
C10	0.0458 (4)	0.7410 (5)	0.3464 (4)	0.0881 (12)	
H10A	−0.0084	0.7303	0.4025	0.132*	
H10B	−0.0169	0.7830	0.2730	0.132*	
H10C	0.1275	0.8084	0.3788	0.132*	
C11	0.7091 (4)	0.3069 (4)	0.2561 (3)	0.0623 (9)	
C12	0.7047 (3)	0.4486 (3)	0.2009 (3)	0.0468 (8)	
C13	0.6475 (3)	0.5778 (4)	0.2417 (2)	0.0492 (8)	
C14	0.5941 (4)	0.5662 (5)	0.3388 (3)	0.0637 (9)	
C15	0.6017 (4)	0.4238 (6)	0.3923 (3)	0.0825 (13)	
H15	0.5667	0.4128	0.4575	0.099*	
C16	0.6584 (4)	0.2998 (5)	0.3527 (3)	0.0823 (12)	
H16	0.6630	0.2069	0.3926	0.099*	
C17	0.7680 (5)	0.1632 (5)	0.2167 (4)	0.0967 (13)	
H17A	0.7070	0.1350	0.1383	0.145*	
H17B	0.7684	0.0817	0.2718	0.145*	
H17C	0.8661	0.1812	0.2150	0.145*	
C18	0.7569 (4)	0.4641 (4)	0.0932 (3)	0.0666 (10)	
H18A	0.8277	0.5468	0.1065	0.080*	
H18B	0.8060	0.3701	0.0830	0.080*	
C19	0.6393 (4)	0.7281 (4)	0.1778 (3)	0.0647 (9)	
H19A	0.6300	0.8108	0.2302	0.078*	
H19B	0.7293	0.7441	0.1578	0.078*	
C20	0.5287 (4)	0.7007 (6)	0.3852 (3)	0.0961 (14)	
H20A	0.6055	0.7695	0.4271	0.144*	
H20B	0.4777	0.6646	0.4388	0.144*	
H20C	0.4614	0.7536	0.3195	0.144*	
O1	0.1514 (2)	0.3950 (3)	−0.00537 (16)	0.0669 (7)	
H1	0.0877	0.3306	−0.0091	0.100*	
O2	0.0586 (2)	0.6779 (3)	0.03506 (18)	0.0690 (7)	
H2	0.0557	0.5913	0.0072	0.103*	
O3	0.6404 (3)	0.4941 (3)	−0.01344 (18)	0.0737 (7)	
H3	0.5828	0.4219	−0.0275	0.110*	
O4	0.5173 (2)	0.7319 (3)	0.07073 (19)	0.0743 (7)	
H4	0.5311	0.6707	0.0223	0.111*	

### Atomic displacement parameters (Å^2^)

**Table:**

	*U*^11^	*U*^22^	*U*^33^	*U*^12^	*U*^13^	*U*^23^
C1	0.069 (2)	0.057 (2)	0.059 (2)	−0.0083 (18)	0.0079 (18)	0.0049 (17)
C2	0.0445 (17)	0.059 (2)	0.0453 (16)	−0.0068 (16)	0.0071 (14)	−0.0018 (15)
C3	0.0408 (17)	0.055 (2)	0.0472 (16)	−0.0070 (15)	0.0069 (14)	−0.0022 (15)
C4	0.0525 (19)	0.074 (2)	0.0494 (17)	−0.0070 (17)	0.0154 (15)	−0.0104 (19)
C5	0.076 (3)	0.110 (4)	0.054 (2)	−0.017 (2)	0.031 (2)	−0.009 (2)
C6	0.088 (3)	0.084 (3)	0.052 (2)	−0.018 (2)	0.0213 (19)	0.012 (2)
C7	0.127 (4)	0.061 (3)	0.099 (3)	−0.002 (2)	0.024 (3)	0.007 (2)
C8	0.056 (2)	0.069 (2)	0.0629 (19)	0.0007 (18)	0.0216 (17)	−0.0039 (18)
C9	0.0588 (19)	0.056 (2)	0.0604 (18)	−0.0109 (16)	0.0156 (16)	−0.0021 (16)
C10	0.088 (3)	0.091 (3)	0.089 (3)	0.003 (2)	0.033 (2)	−0.026 (2)
C11	0.060 (2)	0.058 (2)	0.063 (2)	−0.0066 (17)	0.0096 (17)	0.0018 (18)
C12	0.0415 (17)	0.051 (2)	0.0468 (16)	−0.0064 (14)	0.0112 (14)	−0.0044 (14)
C13	0.0426 (17)	0.056 (2)	0.0446 (16)	−0.0077 (15)	0.0077 (14)	−0.0071 (15)
C14	0.054 (2)	0.086 (3)	0.0487 (17)	−0.007 (2)	0.0122 (16)	−0.017 (2)
C15	0.084 (3)	0.119 (4)	0.051 (2)	−0.019 (3)	0.030 (2)	0.012 (2)
C16	0.094 (3)	0.077 (3)	0.069 (2)	−0.016 (2)	0.016 (2)	0.018 (2)
C17	0.099 (3)	0.060 (3)	0.119 (3)	0.009 (2)	0.016 (2)	−0.006 (2)
C18	0.061 (2)	0.081 (3)	0.0633 (19)	−0.0088 (19)	0.0273 (18)	−0.0105 (18)
C19	0.062 (2)	0.057 (2)	0.067 (2)	−0.0059 (17)	0.0078 (17)	−0.0033 (17)
C20	0.079 (3)	0.131 (4)	0.078 (2)	0.002 (3)	0.024 (2)	−0.039 (3)
O1	0.0763 (16)	0.0749 (17)	0.0522 (12)	−0.0081 (13)	0.0237 (12)	−0.0041 (11)
O2	0.0688 (14)	0.0659 (15)	0.0631 (13)	0.0045 (12)	0.0067 (11)	0.0110 (12)
O3	0.0899 (19)	0.0789 (17)	0.0551 (13)	−0.0164 (14)	0.0266 (13)	−0.0036 (12)
O4	0.0759 (15)	0.0693 (16)	0.0693 (14)	0.0096 (12)	0.0097 (13)	0.0111 (12)

### Geometric parameters (Å, °)

**Table:**

C1—C6	1.384 (5)	C11—C17	1.504 (5)
C1—C2	1.396 (5)	C12—C13	1.400 (4)
C1—C7	1.515 (5)	C12—C18	1.503 (4)
C2—C3	1.402 (5)	C13—C14	1.390 (5)
C2—C8	1.512 (4)	C13—C19	1.500 (5)
C3—C4	1.389 (4)	C14—C15	1.384 (6)
C3—C9	1.503 (4)	C14—C20	1.508 (6)
C4—C5	1.385 (5)	C15—C16	1.355 (6)
C4—C10	1.512 (5)	C15—H15	0.9300
C5—C6	1.358 (6)	C16—H16	0.9300
C5—H5	0.9300	C17—H17A	0.9600
C6—H6	0.9300	C17—H17B	0.9600
C7—H7A	0.9600	C17—H17C	0.9600
C7—H7B	0.9600	C18—O3	1.425 (4)
C7—H7C	0.9600	C18—H18A	0.9700
C8—O1	1.431 (3)	C18—H18B	0.9700
C8—H8A	0.9700	C19—O4	1.432 (3)
C8—H8B	0.9700	C19—H19A	0.9700
C9—O2	1.428 (3)	C19—H19B	0.9700
C9—H9A	0.9700	C20—H20A	0.9600
C9—H9B	0.9700	C20—H20B	0.9600
C10—H10A	0.9600	C20—H20C	0.9600
C10—H10B	0.9600	O1—H1	0.8200
C10—H10C	0.9600	O2—H2	0.8200
C11—C16	1.366 (5)	O3—H3	0.8200
C11—C12	1.391 (4)	O4—H4	0.8200
			
C6—C1—C2	117.6 (3)	C12—C11—C17	123.8 (3)
C6—C1—C7	119.7 (3)	C11—C12—C13	120.9 (3)
C2—C1—C7	122.7 (3)	C11—C12—C18	120.2 (3)
C1—C2—C3	120.3 (3)	C13—C12—C18	118.8 (3)
C1—C2—C8	120.1 (3)	C14—C13—C12	120.2 (3)
C3—C2—C8	119.6 (3)	C14—C13—C19	119.9 (3)
C4—C3—C2	120.7 (3)	C12—C13—C19	119.8 (3)
C4—C3—C9	120.4 (3)	C15—C14—C13	117.2 (3)
C2—C3—C9	119.0 (3)	C15—C14—C20	120.2 (3)
C5—C4—C3	117.9 (3)	C13—C14—C20	122.6 (4)
C5—C4—C10	118.8 (3)	C16—C15—C14	122.1 (3)
C3—C4—C10	123.3 (3)	C16—C15—H15	119.0
C6—C5—C4	121.5 (3)	C14—C15—H15	119.0
C6—C5—H5	119.2	C15—C16—C11	122.1 (4)
C4—C5—H5	119.2	C15—C16—H16	119.0
C5—C6—C1	122.0 (3)	C11—C16—H16	119.0
C5—C6—H6	119.0	C11—C17—H17A	109.5
C1—C6—H6	119.0	C11—C17—H17B	109.5
C1—C7—H7A	109.5	H17A—C17—H17B	109.5
C1—C7—H7B	109.5	C11—C17—H17C	109.5
H7A—C7—H7B	109.5	H17A—C17—H17C	109.5
C1—C7—H7C	109.5	H17B—C17—H17C	109.5
H7A—C7—H7C	109.5	O3—C18—C12	112.6 (3)
H7B—C7—H7C	109.5	O3—C18—H18A	109.1
O1—C8—C2	112.0 (3)	C12—C18—H18A	109.1
O1—C8—H8A	109.2	O3—C18—H18B	109.1
C2—C8—H8A	109.2	C12—C18—H18B	109.1
O1—C8—H8B	109.2	H18A—C18—H18B	107.8
C2—C8—H8B	109.2	O4—C19—C13	111.3 (2)
H8A—C8—H8B	107.9	O4—C19—H19A	109.4
O2—C9—C3	112.7 (2)	C13—C19—H19A	109.4
O2—C9—H9A	109.1	O4—C19—H19B	109.4
C3—C9—H9A	109.1	C13—C19—H19B	109.4
O2—C9—H9B	109.1	H19A—C19—H19B	108.0
C3—C9—H9B	109.1	C14—C20—H20A	109.5
H9A—C9—H9B	107.8	C14—C20—H20B	109.5
C4—C10—H10A	109.5	H20A—C20—H20B	109.5
C4—C10—H10B	109.5	C14—C20—H20C	109.5
H10A—C10—H10B	109.5	H20A—C20—H20C	109.5
C4—C10—H10C	109.5	H20B—C20—H20C	109.5
H10A—C10—H10C	109.5	C8—O1—H1	109.5
H10B—C10—H10C	109.5	C9—O2—H2	109.5
C16—C11—C12	117.4 (3)	C18—O3—H3	109.5
C16—C11—C17	118.7 (3)	C19—O4—H4	109.5
			
C6—C1—C2—C3	0.1 (4)	C16—C11—C12—C13	−1.4 (5)
C7—C1—C2—C3	179.4 (3)	C17—C11—C12—C13	179.6 (3)
C6—C1—C2—C8	−177.9 (3)	C16—C11—C12—C18	−179.2 (3)
C7—C1—C2—C8	1.4 (5)	C17—C11—C12—C18	1.8 (5)
C1—C2—C3—C4	−1.0 (4)	C11—C12—C13—C14	0.1 (4)
C8—C2—C3—C4	177.0 (3)	C18—C12—C13—C14	178.0 (3)
C1—C2—C3—C9	−178.9 (3)	C11—C12—C13—C19	−177.9 (3)
C8—C2—C3—C9	−0.9 (4)	C18—C12—C13—C19	0.0 (4)
C2—C3—C4—C5	1.6 (4)	C12—C13—C14—C15	0.6 (4)
C9—C3—C4—C5	179.5 (3)	C19—C13—C14—C15	178.6 (3)
C2—C3—C4—C10	−178.6 (3)	C12—C13—C14—C20	−178.9 (3)
C9—C3—C4—C10	−0.7 (5)	C19—C13—C14—C20	−0.9 (5)
C3—C4—C5—C6	−1.5 (5)	C13—C14—C15—C16	0.0 (5)
C10—C4—C5—C6	178.7 (4)	C20—C14—C15—C16	179.5 (4)
C4—C5—C6—C1	0.7 (6)	C14—C15—C16—C11	−1.3 (6)
C2—C1—C6—C5	0.0 (5)	C12—C11—C16—C15	2.0 (6)
C7—C1—C6—C5	−179.2 (4)	C17—C11—C16—C15	−179.0 (4)
C1—C2—C8—O1	109.7 (3)	C11—C12—C18—O3	110.9 (3)
C3—C2—C8—O1	−68.3 (4)	C13—C12—C18—O3	−67.0 (4)
C4—C3—C9—O2	−101.5 (3)	C14—C13—C19—O4	−99.6 (3)
C2—C3—C9—O2	76.5 (3)	C12—C13—C19—O4	78.4 (4)

### Hydrogen-bond geometry (Å, °)

**Table:**

*D*—H···*A*	*D*—H	H···*A*	*D*···*A*	*D*—H···*A*
O1—H1···O2^i^	0.82	1.89	2.706 (3)	174
O2—H2···O1	0.82	1.97	2.713 (4)	151
O3—H3···O4^ii^	0.82	1.90	2.709 (4)	167
O4—H4···O3	0.82	1.98	2.717 (4)	150

Symmetry codes: (i) −*x*, *y*−1/2, −*z*; (ii) −*x*+1, *y*−1/2, −*z*.
